# Quality Management Tools on the Stage: Old but New Allies for Rigor and Standardization of Extracellular Vesicle Studies

**DOI:** 10.3389/fbioe.2022.826252

**Published:** 2022-03-10

**Authors:** Giovanna L. Liguori, Annamaria Kisslinger

**Affiliations:** ^1^ Institute of Genetics and Biophysics (IGB), National Research Council (CNR), Naples, Italy; ^2^ Institute of Experimental Endocrinology and Oncology (IEOS), National Research Council (CNR), Naples, Italy

**Keywords:** extracellular vesicles, quality management (QM), optimization, standardization, risk analysis, design of experiments (DoE), standard operating procedure (SOP), failure mode and effect analysis (FMEA)

## Introduction

As early as the second half of the 19th century, Charles Darwin hypothesized as a part of his pangenesis theory that every cell type in the body could generate minute size “gemmules” full of molecules to communicate with other cell types (Darwin, 1868). This seminal intuition fell unnoticed for more than 150 years until contemporary scientists may recognize extracellular vesicles (EVs) in Darwin’s gemmules (Liu and Chen, 2018; Margolis and Sadovsky, 2019; Bergese et al., 2020). Nowadays it has been clearly shown that cells from different organisms, including eukaryotes, both animals (from yeast to mammals) and plants, but also prokaryotic cells, have been demonstrated to release vesicles into the extracellular environment either constitutively or following cell stimulation. EVs have also been isolated from diverse body fluids, including blood, urine, saliva, breast milk, amniotic fluid, cerebrospinal fluid, bile, and semen (Keller et al., 2011; Kalluri and LeBleu, 2020). All EVs are lipid-membrane encapsulated particles filled of cellular content, comprising proteins, metabolites, nucleic acids, lipids, and even entire organelles, some of them specifically sorted and enriched in EV populations with a pattern reflective of cell functions and conditions (Raposo and Stoorvogel, 2013; Yáñez-Mó et al., 2015; Meldolesi, 2018; Van Niel et al., 2018). Very far from representing a tool to eliminate waste material, as hypothesized at the beginning, EVs are able to target specific cells and deliver molecules that induce specific cell response (Van Niel et al., 2018; Kalluri and LeBleu, 2020; Mantile et al., 2020). Therefore, EV-based cell communication has become an extremely intriguing mechanism that attracted a lot of scientists for its great potential in basic as well as applied research.

### Challenges and Initiatives in Extracellular Vesicle Investigation

Due to their interesting features, including high stability in body fluids, capacity to cross the blood brain boundaries, cross-communication among species, EVs have been emerging as key tools for a plethora of applications. Interestingly, EVs are a promising source of new biomarkers for liquid biopsy to be used in the diagnosis, prognosis and treatment of different pathologies, including cancer, immune, inflammatory, cardiovascular and neurodegenerative diseases (Kalluri and LeBleu, 2020; Lin et al., 2020; Tamura et al., 2021; Vozel et al., 2021). Moreover, EVs definitely are a valid alternative to synthetic nanocarriers for drug delivery and for the development of pioneering new therapeutic approaches (Zipkin, 2019; Zarovni et al., 2021).

Thanks also to the outgrowth of new technologies, recent decades have seen a sharp increase in the number of scientific publications focusing on EVs. However, the expanding interest in EV research had also raised some vexing problems, including misleading nomenclature, unknown influence of pre-analytical variables, extreme heterogeneity in the procedures adopted to separate and properly characterize EVs, poor definition of the methodologies themselves, lack of sample quality control and in general of dedicated reference materials and relative standards. As a consequence, this uncertainty slowed down the acceptance of EV potential from scientists of other fields, regulatory agencies, politicians and investors (Roy et al., 2018; Nieuwland et al., 2020; Bazzan et al., 2021). To address these issues, the EV community joined in a common effort and strongly committed itself to rigor and standardization of procedures and reproducibility of results. In 2011, during a meeting in Paris, the International Society for Extracellular Vesicles (ISEV) was founded and in 2012, its official journal, the Journal of Extracellular Vesicles was launched (Lötvall et al., 2012). Since then, ISEV annual meetings and workshops have been regularly organized throughout the world, giving rise to a series of position papers providing guidance on important topics, such as standardization of sample collection and processing (Witwer et al., 2013), RNA analysis (Hill et al., 2013; Mateescu et al., 2017), and diagnostic and therapeutic uses of EVs (Lener et al., 2015; Reiner et al., 2017; Clayton et al., 2018, 2019). In 2014, the ISEV board members provided researchers with the first “Minimal Information for the Study of EVs” (MISEV), a set of biochemical, biophysical and functional standards that should be used to attribute any specific biological cargo or functions to EVs (Lötvall et al., 2014). The MISEV2014 were revised through a community guidance survey (Witwer et al., 2017) and updated as the 382-author “MISEV2018” (Théry et al., 2018), both manuscripts having a strong impact on EV community, as revealed by the extremely high number of citations (more than 1,400 and 2,800 Scopus citations, respectively, by January 2022). MISEV are expected to be further updated according to comments and suggestions of ISEV members, collected through a recent survey (Witwer et al., 2021).

More recently, an ISEV subcommittee specifically devoted to rigor and standardization in EV studies (https://www.isev.org/rigor-standardization) has been created, including several task forces, each one focusing on specific key topics in the field (Nieuwland et al., 2020). Other societies have also been contributing to increase rigor and standardization in EV studies. The International Society on Thrombosis and Hemostasis (ISTH) addressed standardization of EV fluo cytometry (Lacroix et al., 2010; Lacroix et al., 2013; Cointe et al., 2017), and recently collaborated in a transversal working group with ISEV and the International Society for the Advancement of Cytometry (ISAC) to develop guidelines and best practices for EV fluo cytometric experiments, named MIFloCyt-EV (Welsh et al., 2020, 2021; Van Der Pol et al., 2021). Another collaboration has been established between ISEV and the International Society for Cell and Gene Therapy (ISCT) to consider carefully the key issues to address before exploiting the potential of EVs in therapeutic approaches against coronavirus disease-19 (Borger et al., 2020). Meanwhile, several national societies for scientists studying EVs have also been formed and specific EV databases, such as Exocarta (http://www.exocarta.org), Vesiclepedia (Kalra et al., 2012), EVpedia (Kim et al., 2013), exRNA Atlas (Subramanian et al., 2015), and ExoRBase 2.0 (Lai et al., 2022) have been developed to collect the increasing amount of data produced on EV cargo identification. A data depository platform, the EV-track (https://www.evtrack.org; Van Deun et al., 2017), was also created for recording the experimental parameters of EV-related studies providing higher research quality and transparency.

However, the level of adherence to MISEV guidelines and exploitation of additional voluntary online reporting platforms is still unclear. A recent study using a text mining approach on 5,096 accessible EV papers published between 2012 and 2020 has shown that the awareness of investigators to better characterize their EV preparations using a combination of several methods was significantly rised, especially in the studies citing the MISEV position statements (Poupardin et al., 2021). On the other hand, feedback collection on the methods used for EV separation and characterization through more than 600 voluntary ISEV Rigor and Standardization surveys revealed still the lack of sample quality controls, and at the same time the recognized need to give more attention to these topics (Royo et al., 2020). Very recently, the ISEV Board promoted a survey within the ISEV community to understand actual engagement with MISEV, determine how the guidelines could be improved, and define the relationship with other rigor initiatives (Witwer et al., 2021). More than 700 feedbacks were received and analyzed, most of them assessing a strong impact of MISEV on the overall quality of EV studies, but, interestingly about one-third of respondents did not follow the guidelines or did not publish EV studies after MISEV 2018. In a minority of cases, MISEV2018 were perceived too restrictive and long or neglecting key topics. Moreover, the majority of the respondents had not used yet the EV-TRACK platform or were unfamiliar with it (Witwer et al., 2021). From this emerging scenario, the importance of promoting quality culture, improving guideline definition and adherence, and implementing more tools, among which scientist may choose for better and easier standardization of their research, comes fully to light.

### Quality Management at a Glance

The birth of “modern standardization” has been identified at the end of the 18th century with the first Industrial Revolution and the scaling up of production (Kolb and Hoover, 2012; Hellman and Liu, 2013). In the early 1900s, Frederick Winslow Taylor formulated a new approach to factory management, called scientific management, dividing the planning function from the production, focusing on the efficiency and productivity, and introducing pioneer ideas, still valid nowadays, such as employee training and implementation of standardized best practices (Taylor, 2003). In the 1930s, Walter Shewhart of the Bell Telephone Laboratories implemented the Statistical Quality Control of product variables, demonstrating that by eliminating the variation of the process a good standard of end product could be achieved (Shewhart, 1940). At the end of the second World War, thanks to the contribution of Armand Vallin Feigenbaum, Edward Deming, Joseph Moses Juran and Philip Crosby, modern Quality was born, based on the prevention of accidents through the design and implementation of a formal Quality system. Meanwhile, with the very beginning of globalization, the need for standards became internationally recognized. The first international standardizing body with general competence, was the International Standardization Association, created in 1930 and then substituted by the International Organization for Standardization (ISO) founded in 1947. In 1987, ISO published the series of Quality standards which is now known as ISO 9000, widely accepted as “gold standards” in Quality Management (QM), and successively revised in 1994, 2000, 2008, and 2015 (Ziegel and Lamprecht, 1993; Hadjicostas, 2010; ISO, 2019). A QM System is defined as a formalized system that documents processes, procedures and responsibilities, to support the organization activities in meeting their own objectives, customer and regulatory requirements as well as improving their performance on a continuous basis (Ziegel and Lamprecht, 1993; Hadjicostas, 2010; ISO, 2019). In the context of research institutes, a structured approach to QM has a great potential to improve the rigor, reproducibility, reliability and ultimately value and technological transfer potential of scientific research (Bongiovanni et al., 2015; Lanati, 2018; Molinéro-Demilly et al., 2018; Gregory, 2020; Hewera et al., 2020; Liguori and Kisslinger, 2020; Lovrenčić Mikelić, 2020; Hollmann et al., 2021). In addition, the implementation of a QM approach may support the development and execution of research projects, especially in an interdisciplinary, multi-site and high-risk context, providing a roadmap toward improved harmonization and standardization of procedures as well as reliability of results (Dehouck et al., 2019; Liguori and Kisslinger, 2020).

### Quality Management Tools at the Service of EV Research: Fostering Rigor, Standardization, Reproducibility and Technology Transfer

**FIGURE 1:**
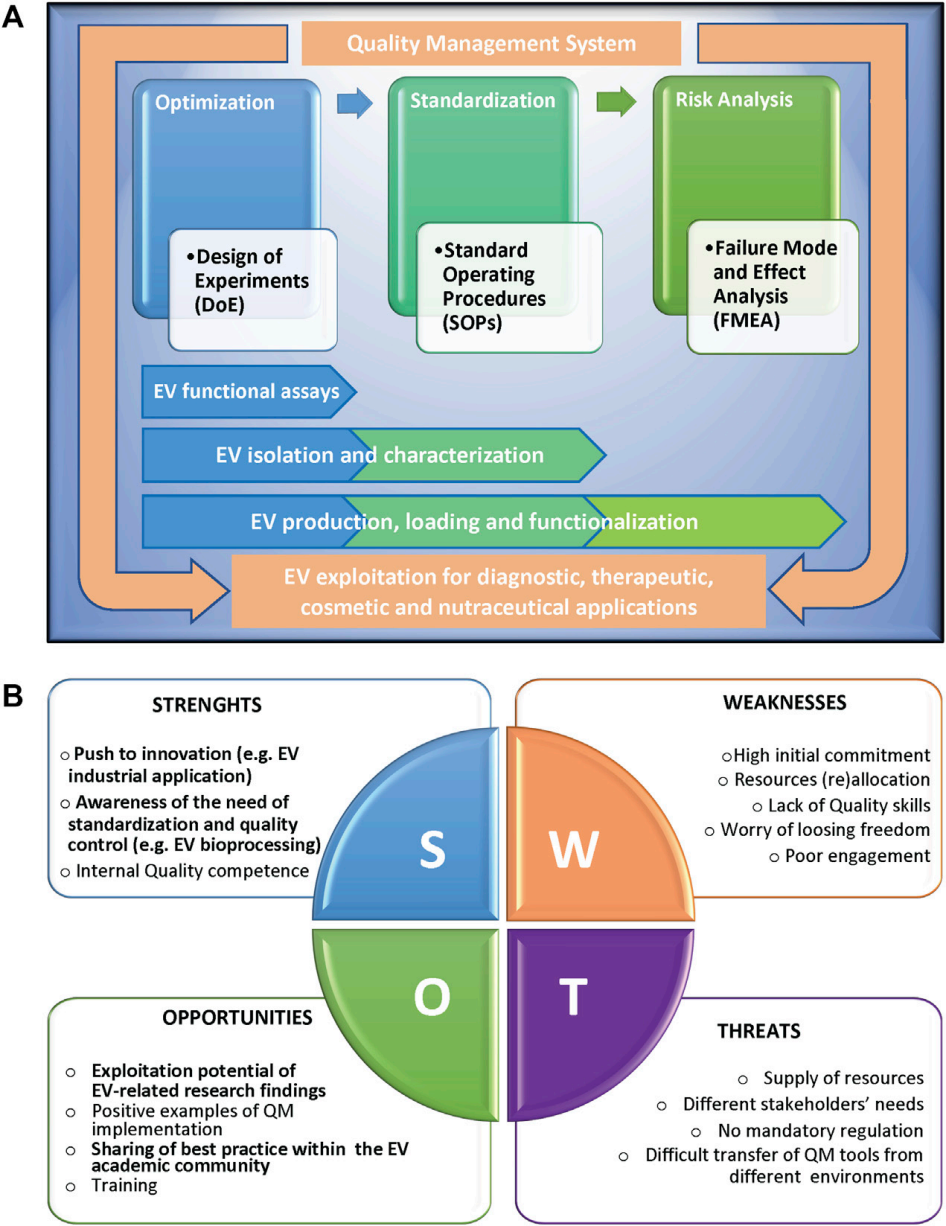
**(A)** Scheme representing the Quality methodologies supporting the different phases of a complex scientific process, from the initial set-up and optimization, to the successive standardization and risk management, with the relative applications to extracellular vesicle (EV) studies. The different methodologies can be applied singularly or can converge in an overall Quality Management System, in compliance with international standards, to support EV exploitation. **(B)** SWOT analysis of the implementation of Quality Management tools in an academic research environment, highlighting the relative Strenghts (S), Weaknesses (W), Opportunities (O), and Threats (T). Factors particularly relevant in the context of EV research are in bold.

The implementation of QM tools can be extremely valid for scientists to successfully overcome the EV challenges (Rovira et al., 2017; Ayers et al., 2019). Here we report a list of methodologies that can be applied in the different phases of a study, from the initial set-up to the full development of a process, and the relative examples of applications for EV research (Figure 1A).

#### Optimization

To find the optimal configurations of variables (factors) which maximize the output(s) of a defined process, traditional experimentation has typically used a one-factor-at-a-time (OFAT) approach, in which every factor is kept constant except for the one under investigation. The OFAT method, however, neglects the complexity of biological processes, that requires instead the simultaneous examination of the factors to be controlled. To overcome this limitation, a Design of Experiments (DoE) statistical approach can be applied to multivariable processes to identify the optimal combination of factors and model their interaction, leading to major benefits in both product performance as well as management of resources (Mandenius and Brundin, 2008; Montgomery, 2012; Weissman and Anderson, 2015; Lipsitz et al., 2016; Myers et al., 2016; Politis et al., 2017; Grangeia et al., 2020). In recent years, the DoE approach is emerging in many fields of scientific research, including cell biology, biochemistry and nanotechnologies (Mancinelli et al., 2015, 2021; Durakovic, 2017; Toms et al., 2017; Lanati, 2018; Narenderan et al., 2019; Papaneophytou, 2019; Xu et al., 2020; Esteban et al., 2021; Papaneophytou et al., 2021; Tavares Luiz et al., 2021). In the EV context, DoE has been successfully implemented to model the effect of different factors, such as time, EV dose, EV type (exosomes or microvesicles) on different parameters related to EV uptake and cargo delivery, fundamental for therapeutic application (Xu et al., 2020; Dave et al., 2021).

#### Standardization

Once a process is optimized, the definition of clear work instructions, such as guidelines and standard operating procedures (SOPs) is highly recommended for controlling activities, assuring reliability and reproducibility of results and last but not least training of new personnel and transfer of competence, and know-how (Hattemer-Apostel, 2001; Gough and Hamrell, 2010; Digilio et al., 2016). SOPs are also very useful for mitigating both health and safety risk as well as instrument damage or erroneous utilization (Akyar, 2012). Definition of SOPs is fundamental for multisite consortia that share results, samples and methodologies as well as for transfer of knowledge and/or technologies to applicative fields. In the EV field, the importance of standard procedures has been specially highlighted for the scalable production of quality standard nanosized EVs from different natural sustainable sources to be used for drug delivery applications (Liguori and Kisslinger, 2020; Buschmann et al., 2021; Paterna et al., 2022).

#### Risk Analysis

Risk management improves the reproducibility of any research process, reducing sources of errors, causing, when intercepted, reworking as well as time and money waste, or, when remain hidden, procedure inaccuracy, high variability, and non-reproducibility of research results. Failure Mode and Effect Analysis (FMEA) is a systematic approach for identifying all possible failures in a design, process, product or service (Tague, 2004; Lanati, 2018). Recently, FMEA has been successfully applied as a tool for risk assessment in biomedical research, biopharmaceutical manufacturing processes, analytical procedures and clinical trials (Zimmermann and Hentschel, 2011; Lee et al., 2017; Mascia et al., 2020; Petretta et al., 2021). Risk analysis is an extremely valid tool to implement in EV basic and even more applicative studies, for the identification and assessment of causes and consequences, and the definition of suitable controls (Reiner et al., 2017).

#### Quality Management System

All the tools described can be used alone or can be implemented in the context of a QM system, in compliance with ISO 9001 standards. Recent examples of the application of QM system in public research institutions have indicated many advantages in terms of governance, control, efficiency, and results (Biasini, 2012; Bongiovanni et al., 2015; Poli et al., 2015; Molinéro-Demilly et al., 2018; Hewera et al., 2020). An ISO-like QM System, slim, flexible and research-oriented has been implemented for the FET Open project entitled VES4US “Extracellular vesicles from a natural source for tailor-made nanomaterials,” conceived since the early beginning with a strong commitment towards Quality culture and principles. The implementation of such QM System supported the project activities, the standardization and sharing of procedures among the different research sites and the achievement of the final objectives (Liguori and Kisslinger, 2020; Adamo et al., 2021; Picciotto et al., 2021).

### Discussion and Future Perspectives

The application of QM to scientific research can positively impact the academic research work by implementing interoperability and activities coordination, improving management of resources and data, increasing reliability and reproducibility of results, and ultimately performance, in term of publications, patents, success in grants applications and/or technology transfer, and overall scientific reputation. As shown by the SWOT analysis in Figure 1B, internal obstacles (weaknesses) to implementation of QM tools include a strong initial commitment, the difficulties to dedicate internal resources, in terms of both money and personnel, the average lack of academic researchers’ specific skills in Quality, and last but not least, the scientists’ worry that such tools might affect research freedom and creativity. Moreover, external threats are the difficulties in supplying resources combined to different needs of stakeholders, in many cases the absence of clear mandatory rules, and the objective obstacles in the transfer of QM models from companies or services to public research environments. All these issues make it hard to motivate scientists to approach Quality culture and implement QM tools. On the other side, positive internal factors (strengths) for QM implementation in academic research are the strong push of several research projects to innovation and to having an impact on society challenges, the growing awareness of standardization relevance, and the availability of internal research staff trained in Quality that can guide QM implementation. Key external factors (opportunities) are the increase of exploitation potential of research findings, of positive examples of QM tools implementation in research institutions, of sharing of best practice and QM models, and of training chances closer to research expectations.

EV community is strongly committed to face important themes such as quality control of EV preparations, standardization of isolation and characterization methodologies, and reproducibility of the results, and then might be very prone to adopt Quality principles and methods in their research activities. Cross contamination among EV research and development and QM might produce new tools and methodologies specifically tailored on the challenges and needs of EV research and researchers, that can really support the field, with the minimum impact on flexibility. The pioneering examples here summarized are showing that the use of general Quality principles and processes, alone or in combinations, is not only a viable option but rather the cornerstone in fostering rigor, standardization and reproducibility to fully access to the high potential of EV findings for medical, industrial and environmental applications.
